# ALKBH5 exacerbates early cardiac damage after radiotherapy for breast cancer via m6A demethylation of TLR4

**DOI:** 10.1515/biol-2025-1184

**Published:** 2025-10-08

**Authors:** Xiaokeya Yasen, Yilinuer Maihesumu, Dilixiati Wusiman, Abudula Aihemaiti, Xirmaimaiti Aishan, Munire Mushajiang

**Affiliations:** Department of Breast Radiotherapy, Cancer Hospital Affiliated to Xinjiang Medical University, 789 Suzhou East Street, Xinshi District, Urumqi, Xinjiang, 830000, China; Department of Tumor Internal Medicine, The First People’s Hospital of Kashgar Prefecture, Kashgar, China; Department of Cardiac Arrhythmia, The First People’s Hospital of Kashgar Prefecture, Kashgar, China

**Keywords:** ALKBH5, m6A modification, TLR4, radiotherapy, myocardial injury, breast cancer

## Abstract

Radiotherapy is an important cancer treatment for breast cancer patients, improving overall survival; however, it can lead to a common complication, radiation-induced heart disease (RIHD). N6-methyladenosine (m6A) RNA modification plays a critical role in the regulation of myocardial function. The aim of this study is to investigate the effect of m6A modification on cardiac injury following radiotherapy for breast cancer. Cardiac dysfunction was assessed by echocardiography and hematoxylin and eosin staining. The expression of ALKBH5 was analyzed by quantitative real-time PCR, western blot, and immunohistochemistry. The underlying mechanism was investigated using methylated RNA immunoprecipitation (MeRIP), RIP, and dual-luciferase reporter assays. The results showed that in RIHD, ALKBH5 expression was upregulated in breast cancer patients after radiotherapy and in RIHD mouse models. ALKBH5 downregulated the m6A modification level of Toll-like receptor 4 (TLR4). Overexpression of TLR4 abolished the inhibitory effect of ALKBH5 silencing on RIHD in mice. In summary, this study revealed a novel regulatory mechanism of ALKBH5-mediated m6A demethylation in RIHD, which could provide a promising therapeutic strategy for cardiac dysfunction following radiotherapy in breast cancer patients.

## Graphical abstract

**Figure:**
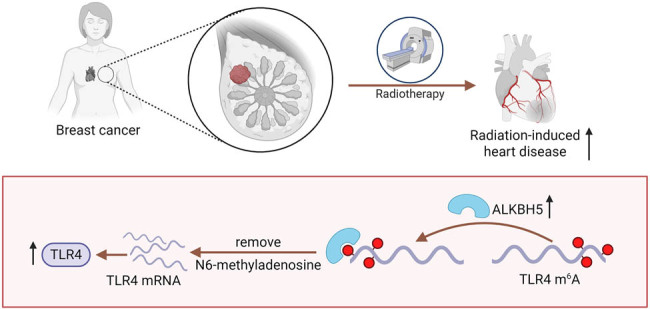

## 1 Introduction

Breast cancer is one of the most common cancers among women worldwide, and its incidence continues to rise [1]. Globally, approximately 2.3 million new cases occur annually, accounting for 11.7% of all cancer cases and resulting in over 685,000 deaths per year [2]. Radiation therapy (radiotherapy, RT) is a critical component of treatment and can significantly improve patient survival rates [3]. However, RT to the left breast or chest wall can lead to radiation-induced heart disease (RIHD), a significant long-term complication characterized by myocardial injury and increased cardiovascular morbidity [4]. Despite advances in RT techniques, the molecular mechanisms underlying RIHD remain poorly understood, hindering the development of targeted therapeutic strategies.

N6-methyladenosine (m6A) RNA modification, the most prevalent internal mRNA modification in eukaryotic cells, regulates gene expression through effects on RNA stability, splicing, and translation [5]. ALKBH5, an m6A demethylase [6], plays a central role in modulating m6A dynamics across diverse biological processes, including cell differentiation, development, stress response, and carcinogenesis, via its demethylation activity [7]. However, its involvement in RIHD remains unexplored. ALKBH5 functions as both an oncogene and a tumor suppressor gene in a context-dependent manner [6,8]. In breast cancer, ALKBH5 is not only implicated in the regulation of cancer stem cells, tumor microenvironment, and drug resistance but also holds potential as a novel therapeutic target and biomarker [9,10]. Nevertheless, its contribution to cardiac pathology following RT remains elusive.

This study unveils a novel regulatory axis linking ALKBH5-mediated m6A demethylation to RIHD in breast cancer patients. We demonstrate that ALKBH5 exacerbates radiation-induced myocardial injury by reducing m6A modification of Toll-like receptor 4 (TLR4), thereby enhancing TLR4 mRNA stability and activating downstream inflammatory signaling pathways. These findings reveal a previously unrecognized mechanism by which epitranscriptomic regulation of RNA metabolism contributes to RT-associated cardiac toxicity, offering a promising therapeutic strategy to mitigate this critical side effect in breast cancer survivors.

## 2 Methods

### 2.1 Clinical sample collection

The study included 30 healthy individuals, 30 breast cancer patients prior to RT, 30 breast cancer patients who had undergone one session of RT, 30 breast cancer patients 1 month post-RT, 30 breast cancer patients 6 months post-RT, and 30 breast cancer patients 12 months post-RT. All participants had left-sided breast tumors, had not received any other treatment modalities, and provided written informed consent. Whole blood samples from all subjects were collected, processed into serum, and stored at −80°C for subsequent experimental analysis.


**Informed consent:** Informed consent has been obtained from all individuals included in this study.
**Ethical approval:** The research related to human use has been complied with all the relevant national regulations, institutional policies, and in accordance with the tenets of the Helsinki Declaration, and has been approved by the Ethics Committee of Cancer Hospital Affiliated to Xinjiang Medical University.

### 2.2 Animal experiment

Female C57BL/6 mice (4–6 weeks old, 20 ± 2 g) were obtained from Nanjing GemPharmatech and housed under controlled environmental conditions (20–26°C temperature, 40–60% humidity, 12 h light/dark cycle). Mice were randomly assigned to nine groups: control, radiation (RT), 1-week post-RT (RT-1W), 2-week post-RT (RT-2W), 4-week post-RT (RT-4W), RT-2W+shNC, RT-2W+shALKBH5, RT-2W+shALKBH5+LV-NC, and RT-2W+shALKBH5+LV-TLR4 (n = 6 per group). The RT group received a single dose of 15 Gy X-ray radiation targeted specifically to the heart [11]. Mice were anesthetized using 2% isoflurane and positioned in a custom-made jig designed for cardiac irradiation. Lead shielding was applied to minimize exposure to non-target organs. Radiation was delivered using a Varian Clinac IX linear accelerator (Varian Medical Systems, Palo Alto, CA, USA) operating at 6 MV with a dose rate of 3 Gy/min. After irradiation, mice were monitored until full recovery from anesthesia. At the end of the study period, mice were euthanized, and hearts were harvested for further analysis.

To investigate the role of ALKBH5 and TLR4 *in vivo*, short hairpin RNA (sh) targeting ALKBH5 (sh-ALKBH5) and a negative control (sh-NC), along with lentivirus-mediated TLR4 overexpression plasmids (LV-TLR4) and a corresponding negative control (LV-NC), were synthesized by GenePharma (Shanghai, China). The sh-ALKBH5 and sh-NC constructs were packaged into lentiviral vectors. Lentivirus (10 μL, 10^9^ PFU/mL) was administered via intravenous injection for 3 consecutive days, followed by cardiac irradiation with X-rays for 2 weeks.


**Ethical approval:** The research related to animal use has been complied with all the relevant national regulations and institutional policies for the care and use of animals, and has been approved by the Ethics Committee of Cancer Hospital Affiliated to Xinjiang Medical University.

### 2.3 Cardiac function assessment

Cardiac function was assessed via echocardiography prior to euthanasia. Echocardiograms were performed using a Vevo 2100 Imaging System (VisualSonics, Toronto, ON, Canada) equipped with a high-resolution 30-MHz probe. Parameters including left ventricular ejection fraction (LVEF), left ventricular posterior wall thickness at end-diastole (LVPWd), and left ventricular posterior wall thickness at end-systole (LVPWs) were measured to evaluate cardiac performance.

### 2.4 Serum cardiac troponin T (cTnT) and B-type natriuretic peptide (BNP) analysis

Serum levels of cTnT and BNP were quantified using commercial ELISA kits in accordance with the manufacturers’ protocols. Human cTnT was measured using the Human cTnT ELISA Kit (ab223860, Abcam, Cambridge, UK), while mouse cTnT was detected using the Mouse cTnT ELISA Kit (MBS2104865, MyBioSource, San Diego, CA, USA). For BNP measurements, human samples were analyzed using the BNP ELISA Kit (ab193694, Abcam), while mouse samples were analyzed using the Mouse BNP ELISA Kit (MBS2510603, MyBioSource). All assays were carried out in duplicate, with absorbance measured at 450 nm using a SpectraMax microplate reader (Molecular Devices, San Jose, CA, USA).

### 2.5 Hematoxylin and eosin (HE) analysis

HE staining was performed using an HE staining kit (G1120, Solarbio, Beijing, China). Briefly, heart tissues were dehydrated through a graded ethanol series, embedded in paraffin, and sectioned into 4 μm-thick slices. After standard dewaxing and washing with distilled water, sections were stained with hematoxylin followed by eosin. Subsequently, the sections were dehydrated, dried, and mounted with neutral resin. Finally, histological images were captured using an optical microscope (Olympus, Tokyo, Japan).

### 2.6 Immunohistochemistry (IHC) analysis

The tumor tissue was collected, fixed, embedded in paraffin wax, and cut into 5 µM sections. After the sections were dewaxed and blocked, they were incubated with the primary antibody against ALKBH5 (ab195377, Abcam) and the secondary antibody. The images were observed under a light microscope (Olympus).

### 2.7 Cell culture and treatment

The rat cardiomyocyte cell line H9C2 (American Type Culture Collection, ATCC) was cultured in high-glucose DMEM (Solarbio) supplemented with 10% FBS (Solarbio), 100 U/mL penicillin, and 100 µg/mL streptomycin at 37°C in a humidified atmosphere with 5% CO₂. For gene manipulation, sh-ALKBH5 or sh-NC plasmids were transfected into H9C2 cells using Lipofectamine 3000 transfection reagent (Invitrogen, Carlsbad, CA, USA).

### 2.8 Quantitative real-time PCR (qPCR)

Total RNA was extracted from designated cell lines or tissue samples using TRIzol reagent (Invitrogen). Subsequently, 1 µg of total RNA was reverse-transcribed into cDNA using a PrimeScript RT Reagent Kit with gDNA Eraser (Takara Bio USA, Inc., Mountain View, CA, USA). Target gene-specific primers were synthesized by Shanghai Sangon Biotech Co., Ltd (Shanghai, China). The mRNA expression levels of target genes were quantified using a QuantStudio5 Real-Time PCR System (Applied Biosystems, Foster City, CA, USA) with SYBR Green-based qPCR. Relative mRNA expression was analyzed using the comparative 2^−∆∆CT^ method, normalized to the housekeeping gene GAPDH.

### 2.9 Methylated RNA immunoprecipitation (MeRIP)

The m6A level of the TLR4 gene was quantified using a GenSeq^®^ m6A MeRIP Kit (CloudSeq, Shanghai, China). Total RNA was extracted from 2 × 10⁷ H9C2 cells and fragmented to an average size of ∼200 nucleotides using RNase III. Magnetic beads were pre-incubated with 2 µL of anti-m6A antibody or isotype control IgG antibody for 1 h on a rotator at 4°C, followed by washing with IP buffer provided in the kit. Subsequently, 200 µL of nuclease-free water containing fragmented RNA was added to the bead-antibody complexes and incubated for 1 h on a rotator at 4°C. After washing with IP buffer, the m6A-enriched RNA was purified, and the enrichment of TLR4 RNA was quantified by qPCR using gene-specific primers.

### 2.10 RNA immunoprecipitation (RIP)

H9C2 cells were washed, centrifuged, and lysed in RIP lysis buffer on ice. After, the cell lysates were incubated with magnetic beads conjugated with anti-ALKBH5 or anti-IgG (ab133470) antibodies at 4℃ overnight. After purification, RNA was extracted from the mixture and analyzed by qPCR.

### 2.11 Dual-luciferase reporter assay

TLR4 promoter sequences (wt) and mutant sequences were separately inserted into the pmiR-GLO vector (Promega, Madison, WI, USA). H9C2 cells were co-transfected with the wt or mutant reporter plasmids along with sh-ALKBH5 or sh-NC plasmids using Lipofectamine 3000 (Invitrogen). The luciferase activities of the aforementioned reporter plasmids were measured according to the protocols provided in the Dual-Luciferase Reporter Assay Kit (Promega).

### 2.12 Western blot

Total proteins from different treatment tissues were extracted using RIPA lysis buffer (Beyotime). After quantification of protein concentration, proteins were separated via 10% SDS-PAGE and transferred to a PVDF membrane. Following transfer, the PVDF membrane was blocked with fast protein-blocking solution (Beyotime) at 37°C for 2 h, incubated overnight at 4°C with primary antibodies at a 1:1,000 dilution, and subsequently incubated with the secondary antibody for 2 h. Band signals were detected using an enhanced chemiluminescence system (Bio-Rad, Hercules, CA, USA). Primary antibodies included: anti-METTL3 (ab195352), anti-METTL14 (ab309096), anti-WTAP, (ab195380), anti-ALKBH5 (ab195377), anti-FTO (ab126605), anti-TLR4 (sc-293072; Santa Cruz, Dallas, TX, USA), anti-p-P38MAPK (4511; Cell Signaling Technology, Danvers, MA, USA), anti-p-ERK1/2 (4370; CST), anti-p-JNK (4668; CST), anti-p-NF-κB (3033; CST), anti-P38MAPK (8690; CST), anti-ERK1/2 (4695; CST), anti-JNK (9252; CST), anti-NF-κB (8242; CST), and anti-β-actin (ab8227).

### 2.13 Bioinformatic analysis

The potential m6A modification sites of TLR4 mRNA were predicted using the online SRAMP database (http://www.cuilab.cn/sramp).

### 2.14 Detection of RNA stability

TLR4 mRNA stability was assessed by qPCR following treatment of H9C2 cells with 5 μg/mL actinomycin D (Act-D; Abmole, Houston, TX, USA) for 1, 2, 4, and 8 h.

### 2.15 Statistical analysis

All experiments were performed in triplicate, with results expressed as the mean value ± standard deviation. Data analysis was conducted using GraphPad Prism software (version 8.3). A Student’s *t*-test was applied to compare differences between two groups, whereas one-way ANOVA was used to assess variations among multiple groups. Normality of all datasets was validated via Shapiro-Wilk testing. The Benjamini-Hochberg FDR method (*q* < 0.05) was employed to correct for multiple comparisons. Correlations between ALKBH5/TLR4 and cTnT expression were analyzed using Pearson’s correlation coefficient. Statistical significance was defined as *p* < 0.05.

## 3 Results

### 3.1 ALKBH5 levels were highly expressed after RT for breast cancer

**Figure 1:**
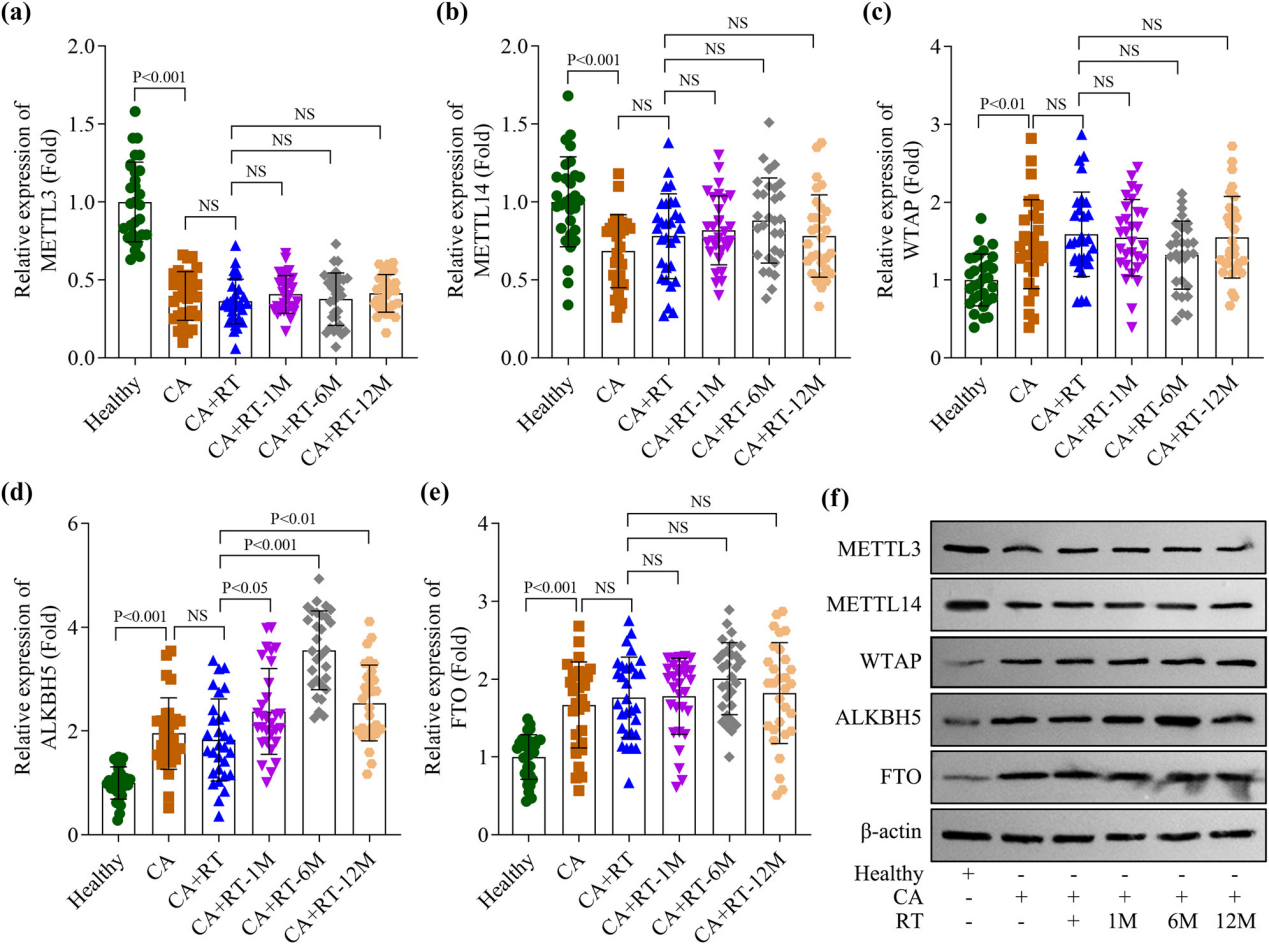
ALKBH5 levels were highly expressed after radiotherapy for breast cancer. (a)–(e) METTL3, METTL14, WTAP, ALKBH5, and FTO levels were detected in healthy subjects, patients with breast cancer before radiotherapy (CA), radiotherapy for once (CA + RT), 1 month after radiotherapy (CA + RT-1M), 6 months after radiotherapy (CA + RT-6M), and 12 months after radiotherapy (CA + RT-12M) using qPCR (*n* = 30 independent biological replicates/group in clinical trials). (f) METTL3, METTL14, WTAP, ALKBH5, and FTO protein levels in the serum of each group were measured using western blotting. All data are expressed as the mean values ± SD. NS: no significance.

To investigate the role of m6A modification in RIHD, we first analyzed the expression levels of methyltransferases (METTL3, METTL14, and WTAP) and demethylases (ALKBH5 and FTO) in serum samples. We observed that METTL3 and METTL14 levels in breast cancer patients were significantly reduced compared to healthy controls, whereas WTAP, ALKBH5, and FTO levels were markedly increased. Notably, no significant differences were observed in METTL3, METTL14, WTAP, or FTO levels between breast cancer patients who received RT and those without treatment. Additionally, ALKBH5 levels remained unchanged in patients undergoing a single session of RT. However, ALKBH5 levels increased progressively at 1, 6, and 12 months post-radiation, with the most pronounced elevation detected at the 6-month timepoint (Figure 1a–e). Western blot analysis further corroborated this finding (Figure 1f). Supplementary analyses revealed a significant increase in cardiac biomarkers, including troponin cTnT and BNP, in breast cancer patients at 1, 6, and 12 months following RT, with the most prominent rise observed at the 6-month timepoint (Figure S1a and b). Importantly, ALKBH5 expression levels exhibited a positive correlation with cTnT expression (Figure S1c). Furthermore, TLR4 expression was significantly upregulated at 1, 6, and 12 months post-radiation, peaking at 6 months (Figure S1d), and demonstrated a positive correlation with cTnT levels (Figure S1e). These findings suggest that ALKBH5 levels are elevated following RT for breast cancer.

### 3.2 RIHD and upregulated ALKBH5 expression in RIHD mice

**Figure 2:**
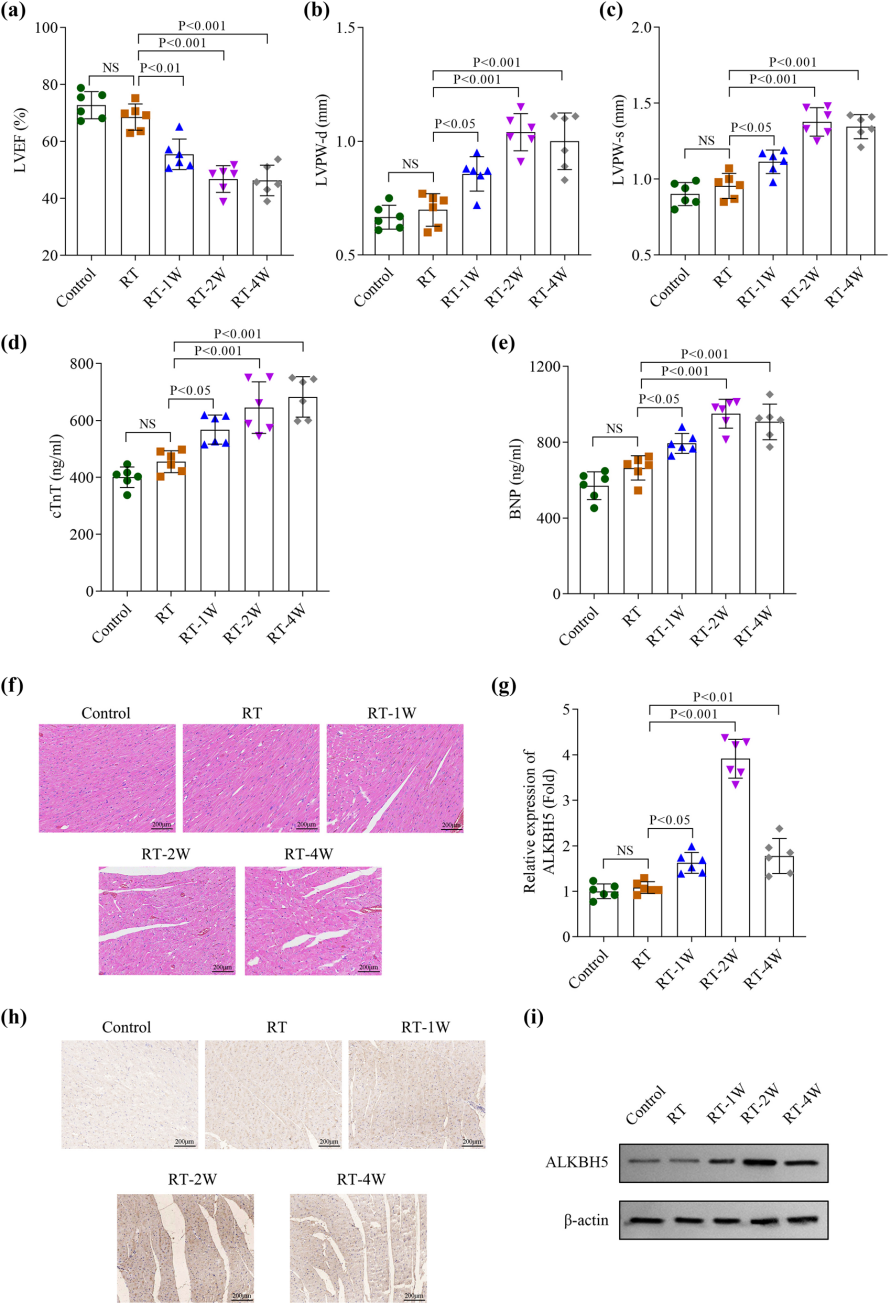
RIHD and upregulated ALKBH5 expression in RIHD mice. (a)–(c) The cardiac function including LVEF, LVPW-d, and LVPW-s was analyzed using the ultrasound imaging system. (d) and (e) The levels of cTnT and BNP (B-type natriuretic peptide) in the serum were detected using an ELISA kit. (f) Myocardial damage was visualized using the HE staining. (g)–(i) The levels of ALKBH5 were examined using qPCR, IHC assays, and western blot in the heart tissues of mice, respectively. All data are expressed as the mean values ± SD. NS: no significance. *n* = 6 independent biological replicates/group in animal experiments.

To validate these clinical findings, we established a murine model of RIHD by irradiating C57BL/6 mice with 15 Gy targeted to the heart. We observed no significant differences in LVEF, LVPW-d, or LVPW-s between mice receiving a single session of RT and the control group. However, LVEF decreased markedly after 1, 2, and 4 weeks of radiation therapy, while LVPW-d and LVPW-s increased significantly after 1, 2, and 4 weeks of radiation, with the most pronounced changes observed at 2 weeks post-radiation (Figure 2a–c). Concurrently, cardiac biomarkers (troponin cTnT and BNP) in mouse serum showed significant increases after 1, 2, and 4 weeks of radiation, with the most prominent elevation at 2 weeks (Figure 2d and e). Histopathological analysis via HE staining revealed inflammatory cell infiltration, disorganized myocardial cells, and disrupted myocardial fibers after 2 and 4 weeks of RT, indicating severe myocardial injury (Figure 2f). Furthermore, qPCR, IHC, and western blot analyses demonstrated that ALKBH5 levels increased progressively from 1 to 4 weeks post-radiation, peaking at 2 weeks (Figure 2g–i). These results confirm that radiation-induced cardiac damage is associated with elevated ALKBH5 expression.

### 3.3 Downregulation of ALKBH5 reversed the myocardium damage induced by radiation in mice

**Figure 3:**
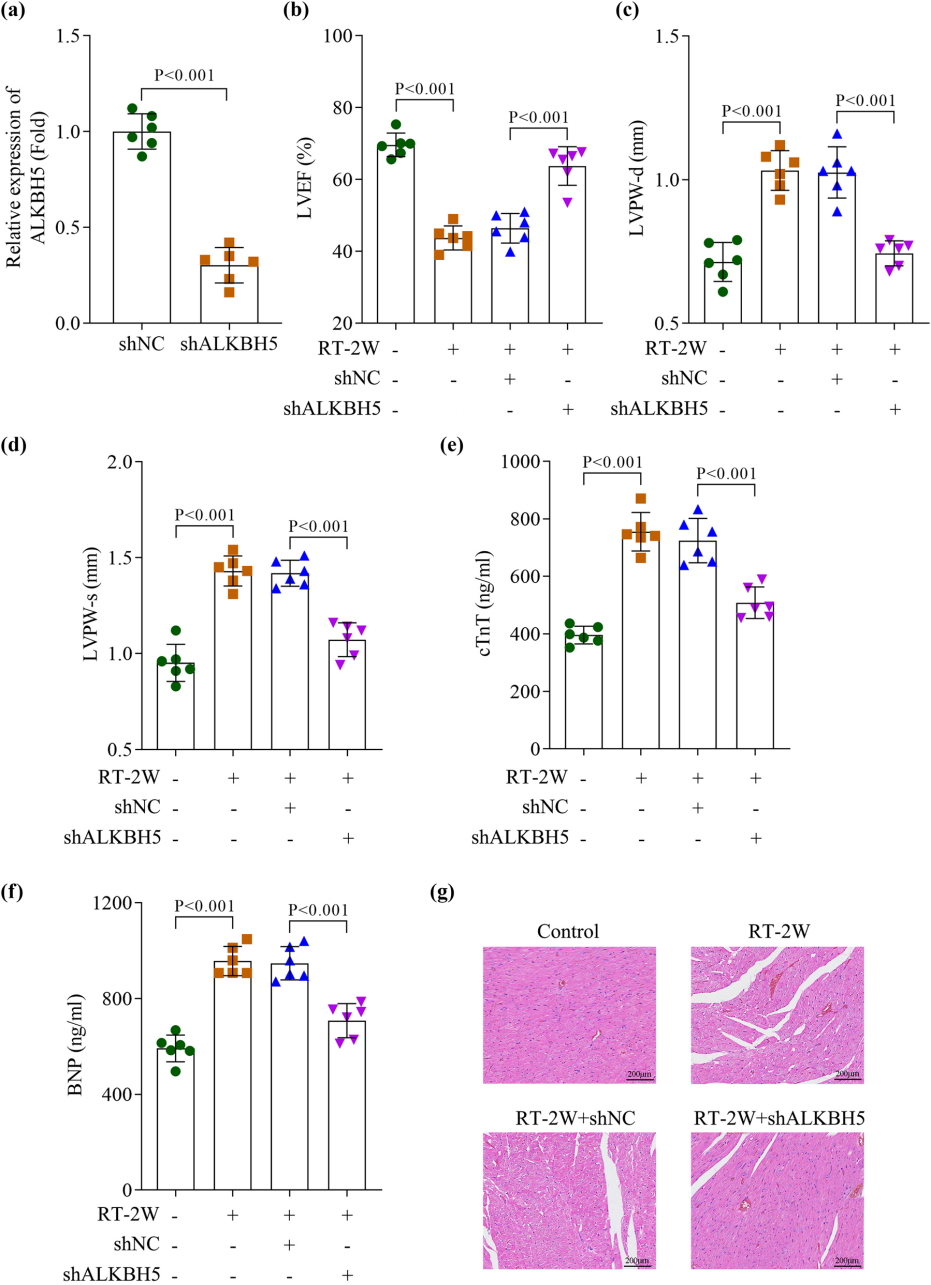
Downregulation of ALKBH5 reversed the myocardium damage induced by radiation in mice. (a) Transfection efficiency was measured by qPCR. (b)–(d) The cardiac function including LVEF, LVPW-d, and LVPW-s was analyzed using the ultrasound imaging system. (e) and (f) The levels of cTnT and BNP (B-type natriuretic peptide) in the serum were detected using an ELISA kit. (g) Myocardial damage was visualized using the HE staining. All data are expressed as the mean values ± SD. *n* = 6 independent biological replicates/group in animal experiments.

To determine whether ALKBH5 contributes to RIHD, we silenced its expression in mice using shRNA (Figure 3a). We observed that 2 weeks of RT induced a significant decrease in LVEF and a marked increase in LVPW-d and LVPW-s, which were mitigated by ALKBH5 knockdown (Figure 3b–d). Similarly, the elevated cardiac biomarkers (troponin cTnT and BNP) in mouse serum following 2 weeks of RT were significantly reduced after ALKBH5 downregulation (Figure 3e and f). Histopathological analysis via HE staining revealed that myocardial damage caused by 2 weeks of RT was alleviated in mice with ALKBH5 knockdown, as evidenced by reduced inflammatory cell infiltration, diminished myocardial fiber breakage, and restored orderly arrangement of cardiomyocytes (Figure 3g). These findings indicate that ALKBH5 silencing attenuates radiation-induced myocardial injury in mice.

### 3.4 TLR4 was the direct target of ALKBH5-mediated m6A demethylation modification

**Figure 4:**
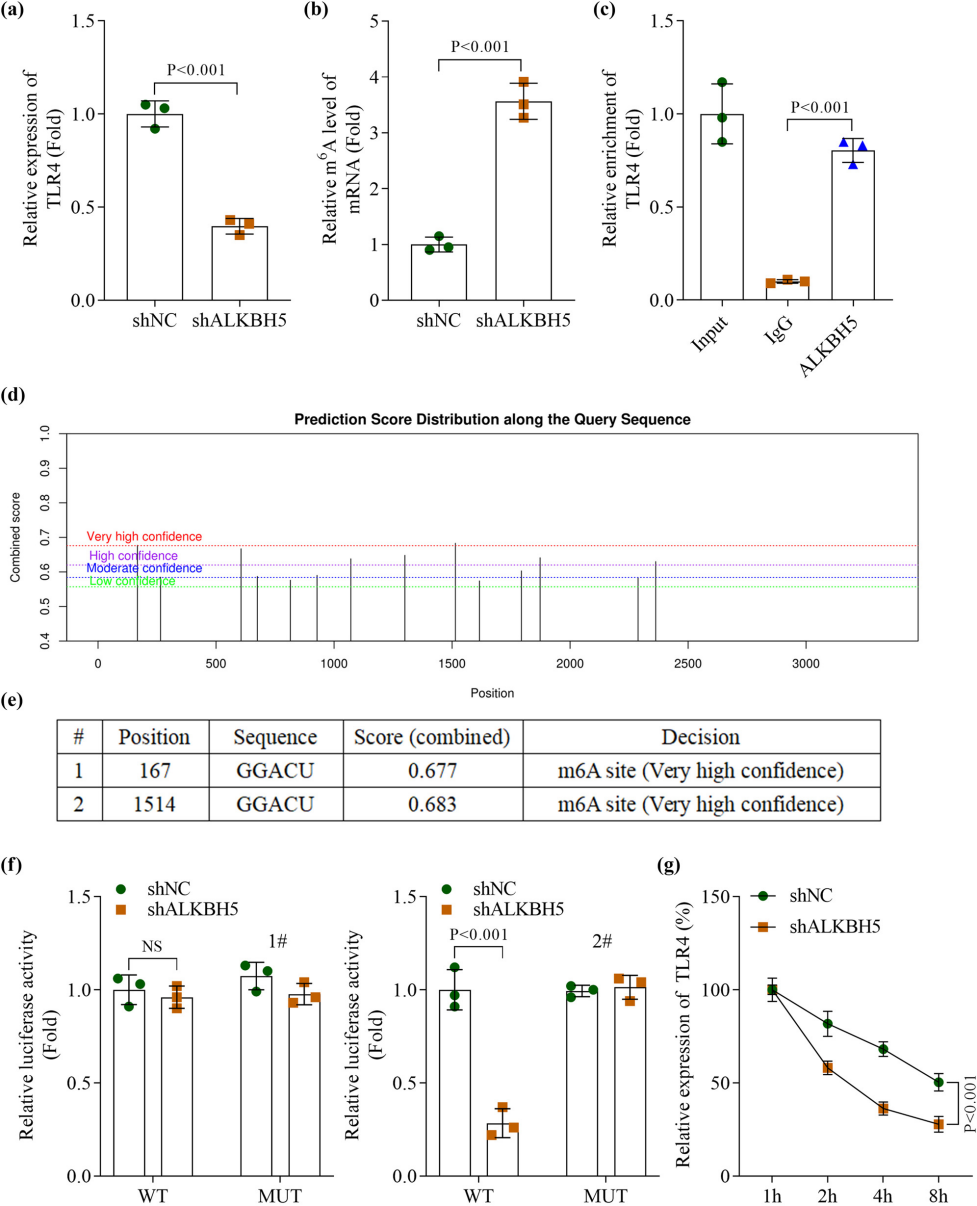
TLR4 was the direct target of ALKBH5-mediated m6A demethylation modification. (a) The mRNA levels of TLR4 were measured using qPCR. (b) The m6A modification level of TLR4 was measured by MeRIP assay. (c) The interaction between TLR4 and ALKBH5 in H9C2 cells was measured by RIP. (d) The potential m6A modification site of TLR4 mRNA was predicted using the online SRAMP database. (e) Selection of mutation sites. (f) Luciferase assay was conducted to examine the binding relationship between TLR4 and ALKBH5. (g) The stability of TLR4 mRNA was measured by qPCR after H9C2 cells were treated with actinomycin D at 1, 2, 4, and 8 h. All data are expressed as the mean values ± SD. NS: no significance. *n* = 3 independent biological replicates/group.

Given the central role of TLR4 in inflammatory signaling, we hypothesized that ALKBH5 regulates TLR4 expression through m6A modification. qPCR revealed that TLR4 mRNA levels were significantly reduced following ALKBH5 knockdown (Figure 4a). ALKBH5 silencing increased m6A levels on TLR4 mRNA (Figure 4b). RIP confirmed the physical interaction between ALKBH5 and TLR4 mRNA (Figure 4c). Bioinformatics analysis using the SRAMP database predicted potential m6A modification sites on TLR4 mRNA (Figure 4d). Specifically, two high-affinity sites (adenine residues at positions 167 and 1,514) were selected for mutagenesis (Figure 4e). Luciferase reporter assays demonstrated that ALKBH5 knockdown significantly reduced luciferase activity in cells transfected with TLR4-wt at the 1514th adenine residue, whereas no significant change was observed in cells expressing the TLR4-mut (Figure 4f). Furthermore, ALKBH5 silencing decreased the stability of TLR4 mRNA (Figure 4g). These data demonstrate that ALKBH5 enhances TLR4 expression by reducing m6A modification levels on its transcript.

### 3.5 Overexpression of TLR4 abolished the inhibitory effect of silenced ALKBH5 on myocardium damage induced by radiation in mice

**Figure 5:**
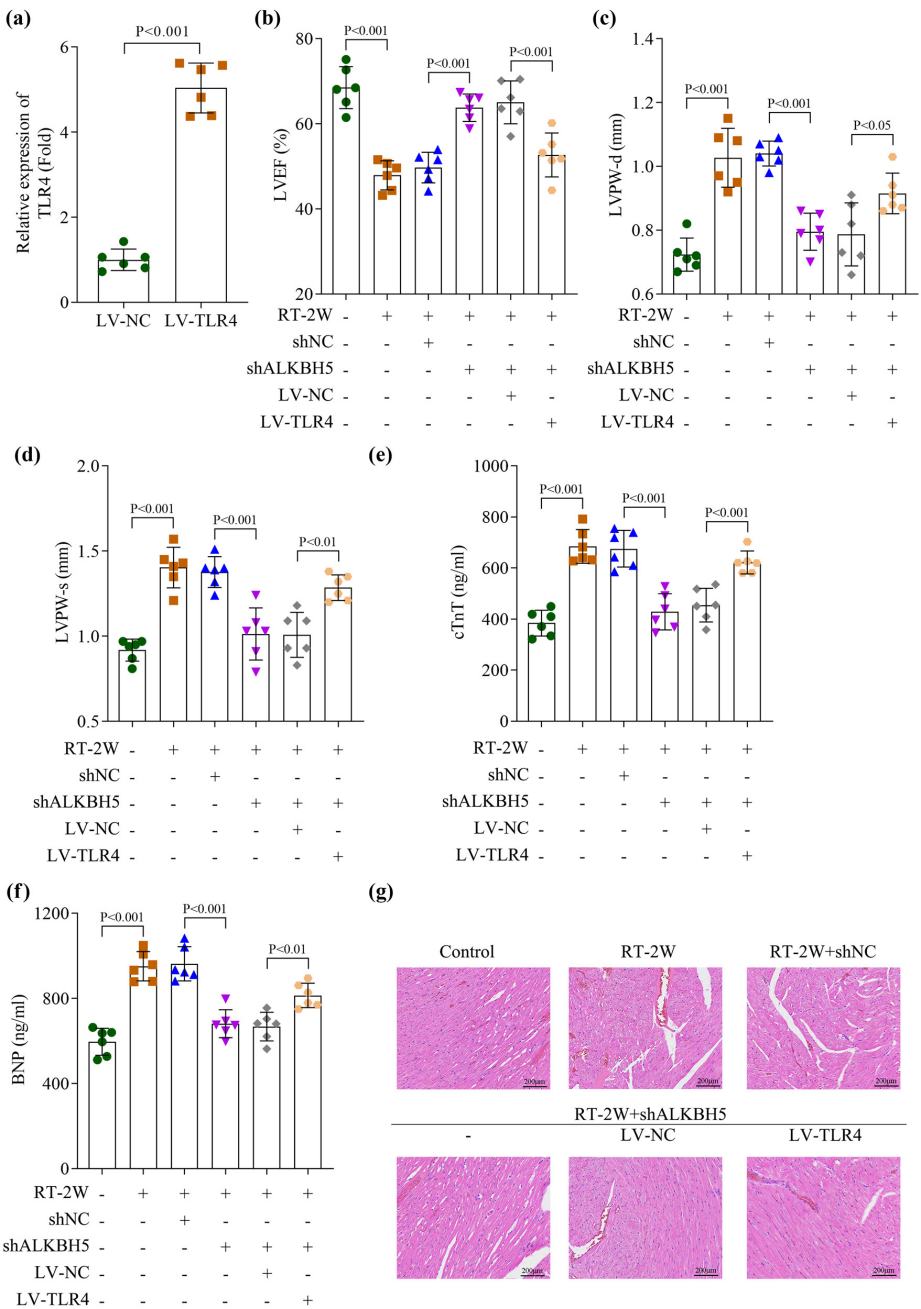
Overexpression of TLR4 abolished the inhibitory effect of silenced ALKBH5 on myocardium damage by radiation in mice induced. (a) Transfection efficiency was measured by qPCR. (b)–(d) The cardiac function including LVEF, LVPW-d, and LVPW-s was analyzed using the ultrasound imaging system. (e) and (f) The levels of cTnT and BNP (B-type natriuretic peptide) in the serum were detected using an ELISA kit. (g) Myocardial damage was visualized using the HE staining. All data are expressed as the mean values ± SD. *n* = 6 independent biological replicates/group in animal experiments.

To confirm TLR4 as a functional mediator of ALKBH5 activity, we overexpressed TLR4 in ALKBH5-knockdown mice (Figure 5a). ALKBH5 silencing significantly elevated LVEF and reduced LVPW-d and LVPW-s after 2 weeks of RT, which was counteracted by TLR4 overexpression (Figure 5b–d). Similarly, the suppression of cardiac biomarkers (cTnT, BNP) in mice following 2 weeks of RT induced by ALKBH5 knockdown was reversed by TLR4 upregulation (Figure 5e and f). Consistently, histopathological analysis via HE staining revealed that ALKBH5 silencing alleviated radiation-induced myocardial injury, whereas TLR4 overexpression abolished the protective effect of ALKBH5 knockdown, as evidenced by restored inflammatory cell infiltration and disrupted myocardial architecture (Figure 5g). To investigate the TLR4-dependent activation of the MAPK/NF-κB signaling pathway, we performed Western blot analysis. Our results demonstrated that TLR4 overexpression reversed the suppression of phosphorylated forms of p-P38MAPK, p-JNK, p-ERK1/2, and p-NF-κB induced by ALKBH5 knockdown (Figure S2), underscoring the role of pro-inflammatory MAPK/NF-κB signaling as a downstream mechanism.

## 4 Discussion

In recent years, RT for breast cancer has become a critical intervention for improving survival rates; however, it is also associated with potential adverse effects, particularly on the heart, including RIHD and long-term cardiovascular risks [12]. To mitigate the cardiotoxic effects of RT, further investigation into the molecular mechanisms of RIHD is essential to identify potential therapeutic targets and strategies. Aberrant regulation of m6A modifications has been strongly linked to various cancers, including breast cancer [13,14]. Moreover, the role of m6A modification in cardiac injury has garnered significant attention [15]. m6A modification influences cardiac injury progression by modulating cardiomyocyte survival, apoptosis, autophagy, and inflammatory responses [16]. Given the critical roles of m6A modification in both oncogenesis and myocardial damage, therapeutic strategies targeting m6A-related proteins are increasingly being explored. Our findings revealed altered expression patterns of m6A methyltransferases (METTL3, METTL14, and WTAP) and demethylases (ALKBH5 and FTO) in the serum of healthy individuals vs breast cancer patients. Notably, among these enzymes, only ALKBH5 levels were significantly elevated in breast cancer patients undergoing RT for 1–12 months; other m6A-modification-associated enzymes remained unaffected by radiation exposure. Previous studies have demonstrated that high-dose radiation (>5 Gy) can induce heart disease in patients receiving RT [17]. Collectively, these findings provide a strong rationale for our investigation into the role of ALKBH5 in radiation-induced myocardial injury.

Radiation exposure impacts multiple aspects of cardiac physiology, including the coronary arteries, myocardium, pericardium, and valves [18,19]. Studies have shown that high-dose radiation exposure in mice typically leads to reduced LVEF and elevated expression of biomarkers indicative of myocardial injury [20,21]. Similarly, Xu et al. found that high-dose ionizing radiation induces cardiac dysfunction and structural remodeling in mice, characterized by decreased LVEF and LVPW-s, along with increased LVID-s [22]. In our study, we established a RIHD model and observed that LVEF decreased while LVPW-d, LVPW-s, cTnT, and BNP increased in mice after 1 week of radiation exposure, with the most pronounced changes at 2 weeks post-irradiation. Pathological analysis further confirmed severe myocardial injury in mice at this timepoint. Notably, ALKBH5 RNA levels and protein expression were significantly elevated in mice following 2 weeks of radiation. However, ALKBH5 knockdown reversed the radiation-induced myocardial damage. These findings suggest that ALKBH5 plays a regulatory role in radiation-induced cardiac injury.

Subsequently, we conducted *in vitro* experiments to investigate the mechanism by which ALKBH5 regulates myocardial function. We found that ALKBH5, as an m6A demethylase, reduces the m6A modification level of TLR4, thereby increasing TLR4 expression. TLR4, a member of the TLR family, is well-established as the primary receptor for bacterial lipopolysaccharide [23]. Beyond its critical role in infectious diseases, TLR4 profoundly influences cancer initiation, progression, metastasis, and therapeutic response [24], particularly in breast cancer [25]. In breast cancer cells, TLR4 is significantly overexpressed, and this overexpression is closely associated with tumor aggressiveness, disease progression, and poor patient prognosis [26]. Notably, TLR4 undergoes post-transcriptional regulation via m6A modification, which alters its functional activity, modulates downstream signaling pathways, and thereby impacts immune response [27] and oncogenesis [28]. Additionally, TLR4 plays a pivotal role in radiation-induced cardiac injury [29], mediating processes such as inflammatory responses, oxidative stress, cardiac remodeling, and immune modulation [30]. In our study, we observed that TLR4 upregulation counteracted the protective effect of ALKBH5 knockdown on radiation-induced myocardial injury in mice, confirming that ALKBH5 contributes to radiation-induced cardiac damage through TLR4 overexpression. Furthermore, we investigated the role of TLR-mediated signaling pathways in cardiac injury. A study has demonstrated that excessive activation of the TLR4/MAPKs/NF-κB pathway serves as a critical molecular mechanism exacerbating cardiac toxicity [31]. Here we provide evidence that TLR4 overexpression effectively reverses the suppression of MAPKs/NF-κB pathway activation induced by ALKBH5 knockdown. These results demonstrate that TLR4 hyperactivation contributes to cardiac dysfunction and suggest ALKBH5 as a potential regulatory factor in this pathway.

Several limitations should be acknowledged in this study. The use of H9C2 cells and mouse models may not fully replicate human cardiac responses due to species-specific differences. Future work should validate findings in human primary cardiomyocytes and explore additional pathways beyond MAPK/NF-κB. While the mouse model successfully mimicked radiation-induced cardiac injury, interspecies differences in cardiac pathophysiology may limit the direct translation of these results to humans. Moreover, broader clinical studies are needed to assess generalizability across diverse populations and support personalized therapies.

In conclusion, our study demonstrates that ALKBH5 plays a critical role in radiation-induced cardiac injury. ALKBH5 enhances the stability of TLR4 mRNA by reducing its m6A methylation levels, thereby promoting the progression of radiation-induced myocardial damage. These findings not only identify ALKBH5 as a potential therapeutic target for mitigating cardiac injury in breast cancer patients undergoing RT but also provide a comprehensive framework for exploring the role of m6A modifications in the adverse effects of cancer RT.

## Supplementary Material

Supplementary Figure
