# Supplementary material for: ALKBH5 exacerbates early cardiac damage after radiotherapy for breast cancer via m6A demethylation of TLR4
Source: Open Life Sci. 2025 Oct 8;20(1):20251184. doi: 10.1515/biol-2025-1184 (PMC12514778; doi:10.1515/biol-2025-1184)
Supplement: Supplementary Figure [file biol-2025-1184-sm.pdf]

Supplementary material

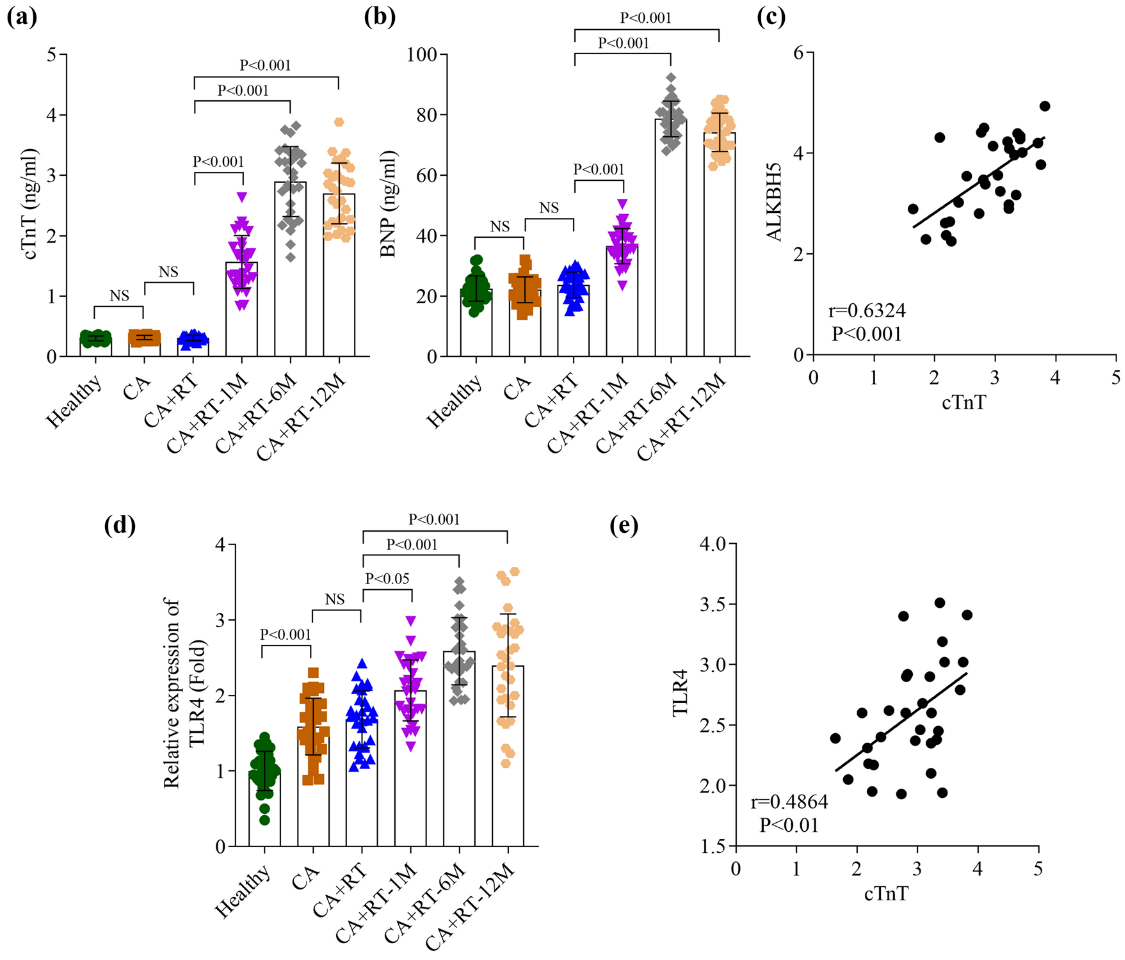

Figure S1: The correlation analysis between ALKBH5/TLR4 levels and cTnT or BNP. (a) and (b) The levels of cTnT (cardiac troponin T) and BNP (B-type natriuretic peptide) in the serum of subjects were detected using an ELISA kit. (c) The association between ALKBH5 and cTnT expression was assessed using Pearson's correlation analysis. (d) TLR4 levels were detected using qPCR. (e) The association between TLR4 and cTnT expression was assessed using Pearson's correlation analysis. All data are expressed as the means ± SD. NS: no significance. (*n* = 30 independent biological replicates/group in clinical trials).

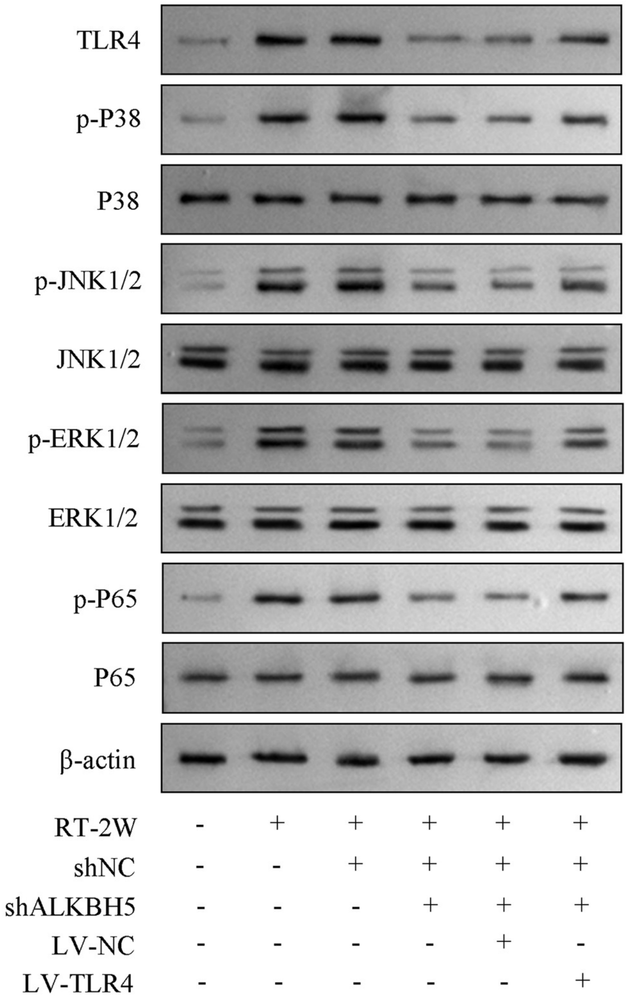

Figure S2: Western blot was performed to investigate the regulatory effects of ALKBH5 and TLR4 on the MAPK/NF-κB signaling pathway.
